# Estimating the Fraction of Hepatocellular Carcinoma and Decompensated Cirrhosis Attributable to Viral Hepatitis in England Using Electronic Health Records and Case‐Notes Review

**DOI:** 10.1111/jvh.70228

**Published:** 2026-09-21

**Authors:** David Etoori, Annabel Powell, Paul Trembling, Jennifer Plunkett, Ashley Brown, Maud Lemoine, Stephen Ryder, Sarah Montague, Eleanor Pilsworth, Anthony Bains, Laura Harrison, Michael Pavlides, Ahmed Elsharkawy, Caroline Sabin, Monica Desai, Ruth Simmons

**Affiliations:** ^1^ Centre for Clinical Research, Epidemiology, Modelling and Evaluation (CREME) Institute for Global Health, University College London UK; ^2^ National Institute for Health and Care Research (NIHR) Health Protection Research Unit (HPRU) in Blood‐Borne and Sexually Transmitted Infections at UCL in Partnership With the UK Health Security Agency (UKHSA) London UK; ^3^ Blood Safety, Hepatitis, Sexually Transmitted Infections and HIV Division UK Health Security Agency London UK; ^4^ Division of Medicine, Institute for Liver and Digestive Health University College London, Royal Free Hospital London UK; ^5^ Imperial College Healthcare NHS Trust St Mary's Hospital London UK; ^6^ Nottingham University Hospitals NHS Trust Nottingham UK; ^7^ NIHR Nottingham Biomedical Research Centre at Nottingham University Hospitals NHS Trust Nottingham UK; ^8^ Sheffield Teaching Hospitals NHS Foundation Trust Sheffield UK; ^9^ NIHR Biomedical Research Centre Oxford University Hospitals NHS Foundation Trust and University of Oxford Oxford UK; ^10^ NIHR Biomedical Research Centre at University Hospitals Birmingham NHS Foundation Trust and University of Birmingham Birmingham UK

## Abstract

Viral hepatitis mortality is an impact target required to evidence the elimination of viral hepatitis with a combined mortality rate of less than or equal to six deaths per 100,000 population. Within England, routine healthcare data linked with death registrations are used; however, where these data are not available, the World Health Organization (WHO) recommends overlaying the attributable fraction (AF) over the mortality envelope for decompensated cirrhosis (DC) and hepatocellular carcinoma (HCC). We aimed to estimate viral hepatitis mortality using the attributable fraction and compared it to surveillance methods. Six acute NHS trusts participated across England and reviewed the case records of individuals identified retrospectively up to 31 December 2023 through hospital episode statistics to confirm the diagnosis and complete a proforma on possible exposures and likely cause of DC/HCC. Overall, 563 individuals were identified, 390 for DC review with 127 (32.6%) confirmed and 173 for HCC review with 157 (91.8%) confirmed. The AF for DC was 0.008 for HCV and 0.016 for HBV, equivalent to a crude mortality rate of 0.09 per 100,000 population and 0.17 per 100,000 population, respectively. For HCC 0.1806 for HCV and 0.0645 for HBV, equivalent to 0.85 per 100,000 population and 0.30 per 100,000 population respectively. These results are comparable to applying the attributable fraction methodology to linking healthcare data sets and demonstrate that England continues to meet the WHO impact target.

## 1 Background

The World Health Organization (WHO) estimates that globally 287 million people are living with viral hepatitis, 240 million with hepatitis B and 47 million with hepatitis C, with approximately 1.8 million new infections of HBV and HCV a year. Additionally, the WHO reported that in 2024 an estimated 1.1 million people died from HBV and 240,000 from HCV related causes, primarily cirrhosis and hepatocellular carcinoma (HCC) [1]. Hepatocellular carcinoma represents a major cause of mortality among people with viral hepatitis, with a substantial proportion of cases attributable to HBV and HCV globally, alongside other risk factors, as highlighted in a recently updated, systematic review and meta‐analysis [2].

Based on testing data and the 2021 census, 270,000 people were estimated to be living with chronic hepatitis B in England, with less than half of those having had a positive hepatitis B test. For HCV, 2024 prevalence estimates were 50,200, a reduction of 61.1% from an estimated 129,000 in 2015 [3]. This reduction is associated with improved access to curative direct‐acting antivirals (DAAs) through testing and treatment initiatives led by NHS England in partnership with NHS trusts, the pharmaceutical industry and lived‐experience and third‐sector partners [3].

Reductions in mortality associated with HCC and end‐stage liver disease (ESLD) have been observed among people with HCV within England, whereas mortality associated with HBV has remained relatively stable over time. Mortality is one of the impact indicators used by the WHO to assess progress towards eliminating viral hepatitis as a public health threat. The WHO target is an annual mortality rate of no more than six hepatitis B‐ and hepatitis C‐related deaths per 100,000 population, replacing earlier disease‐specific targets of four per 100,000 for hepatitis B and two per 100,000 for hepatitis C. Powell et al. [4], reported that England has achieved this target, estimating that mortality from ESLD and HCC attributable to HBV and/or HCV ranged from 0.54 to 1.14 deaths per 100,000 population in 2023. These estimates were derived using death registrations alone (lower bound) and linked death registrations, hepatitis surveillance records and hospital data (upper bound). The use of linked routine healthcare datasets was intended to account for underreporting of viral hepatitis on death certificates, which has been estimated to range from 31% to 59% among people with HCV who died of liver disease [5, 6, 7, 8, 9].

An alternative approach recommended by the WHO to estimate hepatitis‐related mortality where national surveillance data may not be reliable is to overlay the attributable fraction—the proportion of individuals with decompensated cirrhosis and HCC attributable to hepatitis B or C—over the number of deaths from decompensated cirrhosis and HCC [10, 11, 12]. The primary aim of this study is to estimate national mortality from decompensated cirrhosis and HCC attributable to viral hepatitis (HBV and HCV) using the WHO attributable fraction methodology through case‐note review at sentinel hepatology centres in England. Secondary aims were to assess the feasibility of implementing the WHO protocol through a multicentre, sentinel case‐note review in England and compare the resulting mortality estimates with those derived from established, surveillance‐based methods.

## 2 Methods

### 2.1 Study Population

We conducted a multicentre sentinel case‐note review across six England acute NHS trusts to estimate the fraction of cirrhosis and HCC attributable to chronic HBV or HCV. The methods were developed using the WHO protocol for surveillance of the fraction of cirrhosis and HCC attributable to viral hepatitis in clinical centres of excellence [11]. Participating sites agreed to undertake a case‐note review and included specialist hepatology and gastroenterology units, two from London (St Mary's Hospital and the Royal Free Hospital) and four outside London (Royal Hallamshire Hospital Sheffield, Nottingham University Hospital, Queen Elizabeth Hospital Birmingham, John Radcliffe Hospital, Oxford). Collectively, these centres provide broad geographical representation across England and are major tertiary hepatology referral centres serving populations with diverse demographic characteristics and varying risk profiles for viral hepatitis, including large urban populations and regional referral networks for advanced liver disease.

### 2.2 Case Definitions

Decompensated cirrhosis (DC) and hepatocellular carcinoma (HCC) were the outcomes of interest in this study. However, because DC is challenging to identify reliably from routine administrative data alone, a broader set of ICD‐10 codes capturing end‐stage liver disease (ESLD) and its complications was used to identify potential cases for case‐note review. Final inclusion required confirmation of DC through a detailed review of the clinical record.

ICD‐10 codes were used to define HCC (C22.0) and decompensated cirrhosis (I850, I983, K704, K720, K721, K729, K767 and R18).

As part of the study, we also assessed how accurately these codes classified cases by comparing ICD‐10–based identification with diagnoses confirmed through detailed case‐note review.

### 2.3 Data Collection

Individuals with decompensated cirrhosis and/or HCC, attending the participating centres prior to 31 December 2023 were identified through NHS England hospital episode statistics (HES) of all contacts with NHS hospitals in England, or the emergency care data set (ECDS) of attendances at NHS emergency care services in England. Individuals were identified retrospectively, beginning with those recorded on 31 December 2023 and moving backward through time sequentially until the sample size was met for each site; this occurred within six months for all sites for HCC and two months for DC.

The sample size was estimated using the expected proportion of either decompensated cirrhosis or HCC with 5% absolute precision using the following formula, where *z* is the z‐score corresponding to the confidence level (1.96 for 95% confidence), p is the expected proportion, and *d* is the absolute precision (0.05):

$$\frac{Z^{2}p\left(1-p\right)}{d^{2}}$$

$$n=\frac{{\left(1.96\right)}^{2}\times 0.11\;x\;0.89}{{\left(0.05\right)}^{2}}=\frac{3.84\times 0.098}{0.0025}=151$$

We estimated that we would require 150 individuals with cirrhosis and 150 with HCC, resulting in 25 individuals with decompensated cirrhosis and 25 individuals with HCC per acute trust. Sample size calculations were performed separately for decompensated cirrhosis and HCC.

Individual lists were shared with sites for a case‐note review using a proforma. Sites were asked to confirm the diagnosis of decompensated cirrhosis and/or HCC, and to indicate the possible exposures and likely cause. The likely cause was assigned by the reviewing clinician based on available clinical information within the patient record. To promote consistency across sites, a data extraction report form was used (see Data S1). Information was collected on demographics; liver disease status; HBV and HCV markers; alcohol‐related liver disease; metabolic dysfunction‐associated steatotic liver disease (MASLD, previously non‐alcohol fatty liver disease (NAFLD)); autoimmune/metabolic causes; HIV status; diabetes; body mass index (BMI, where available); imaging, laboratory and histopathology results; and admission details. Additional records were shared with sites if decompensated cirrhosis or HCC was not confirmed to ensure sites reached the defined sample size.

Data from each site were collated. Individuals were excluded if decompensated cirrhosis or HCC was not confirmed, or if they were missing a possible exposure and/or had indeterminate HBV/HCV results.

HBV infection status was determined as an HBsAg positive result and HCV infection status by detectable HCV RNA and/or positive HCV antigen confirmation (active infection).

Individuals positive for both HBV and HCV were reviewed to determine the likely cause, based on disease status, evidence of treatment and disease stage at treatment initiation. Individuals with both decompensated cirrhosis and HCC were coded as HCC.

### 2.4 Statistical Analysis

All summary statistics for data are presented as absolute values with percentages, where data were reported.

The proportion of individuals with decompensated cirrhosis and HCC who tested positive for HBV or HCV was calculated to estimate the attributable fraction [13]. The attributable mortality is then calculated as the attributable fraction multiplied by the number of deaths due to decompensated cirrhosis or HCC. Deaths due to decompensated cirrhosis or HCC are identified using ONS death registrations, which include all deaths registered in England regardless of the deceased's usual residence, using the same ICD10 codes for cause of death.

Mortality rates per 100,000 population are calculated as the attributable mortality divided by the mid‐year population estimates multiplied by 100,000.

#### 2.4.1 Estimating HCV‐Related Mortality From HCC Per 100,000 Population

$$\mathrm{AF}=\text{Proportion exposed}\;\left(\mathrm{HCV}/\mathrm{HCC}\right)$$

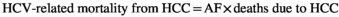




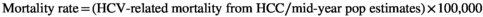




### 2.5 Sensitivity Analysis

We compared mortality rates estimated through the case‐note review with two alternative methods using routinely available healthcare datasets. A summary of the data sources, linkage strategy, case definitions and mortality estimation approach used for each method is provided in Table S1.

#### 2.5.1 Method A: Surveillance‐Linked Mortality Estimation

Method A estimated viral hepatitis‐related mortality using linked surveillance and mortality datasets. Deaths from ESLD/HCC were identified through death registrations and linked to laboratory‐confirmed HBV/HCV diagnoses and hospital admission records [4].

#### 2.5.2 Method B: Administrative Data Attributable Fraction Estimation

Method B estimated viral hepatitis‐related mortality using an attributable fraction approach applied to routine administrative healthcare data. The attributable fraction was calculated as the proportion of individuals with HCC or DC identified in hospital datasets who also had evidence of HBV or HCV infection.

Mortality rates per 100,000 population are calculated as the number of deaths divided by the mid‐year population estimates multiplied by 100,000.

### 2.6 Ethics

This study received ethical approval from the UK Health Security Agency Research and Public Health Practice Ethics and Governance Group (UKHSA REGG—reference NR0397) and from the UKHSA Caldicott Panel (reference CAP‐2024‐20).

## 3 Results

A total of 300 individuals were included on the lists that were initially shared with sites for the case‐note review: 150 with ICD‐10 codes indicating cirrhosis (25 per site) and 150 with ICD‐10 codes indicating HCC (25 per site). Due to the low classification confirmation of decompensated cirrhosis resulting from the sensitivity of some of the ICD‐10 codes, and to ensure each site reached their sample size, further lists including totals of 240 and 23 individuals were shared for decompensated cirrhosis and HCC, respectively. Overall, sites received information on 563 individuals: 390 for cirrhosis review with 127 (32.6%) confirmed and 173 for HCC review with 157 (90.8%) confirmed.

### 3.1 Attributable Fraction Estimates of HCC due to Viral Hepatitis

The distribution of individuals with confirmed HCC (157) can be seen in Table 1. Most had a likely cause of HCC reported (155; 98.7%) and 135 (87.1%) had been screened for viral hepatitis. The median age was 74 years (interquartile range (IQR): 67–79), 77.5% (100/129) were male and 85.7% (108/126) were of White ethnic origin. HCV was reported as the likely cause of HCC for 18.1% (28/155) of individuals and HBV for 6.5% (10/155) of individuals, resulting in an estimated attributable fraction of 0.18 for HCV and 0.06 for HBV (Table 2).

**TABLE 1:** Demographics of individuals with hepatocellular carcinoma (HCC) attending six acute NHS trusts in England in 2024.

	*N*	%
HCC shared	173	100
HCC confirmed	157	90.8
HCC with likely cause	155	98.7
**Sex**		
Male	100	77.5
Female	29	22.5
Unknown	26	
**Median age**	74 years (IQR: 67–79)	
**Ethnic group**		
Asian	5	4.0
Black	3	2.4
White	108	85.7
Other	10	7.9
Unknown	29	
**HCC diagnosis tool**		
CT	43	22.6
Biopsy	25	13.2
MRI	73	38.4
AFP	8	4.2
Radiology	25	13.2
Histology	14	7.4
USS	1	0.5
Biochem	1	0.5
**Site**		
Sheffield	25	16.1
Birmingham	26	16.8
Oxford	26	16.8
Nottingham	26	16.8
London	52	33.5
**HBV tests**		
HBV positive	11	8.1
HBV negative	124	91.9
**HCV tests**		
HCV Ab negative	102	75.6
HCV Ab positive	33	24.4
HCV RNA positive	2	1.5
HCV Ag core positive	2	1.5
**Likely cause of HCC**		
HBV	10	6.5
*HBV only*	7	4.5
*With HCV*	1	0.6
*With HDV*	1	0.6
*With MASLD*	1	0.6
HCV	28	18.1
*HCV only*	15	9.7
*With alcohol*	8	5.2
*With MASLD*	5	3.2
MASLD	69	44.5
Alcohol related	41	26.5
Other	13	8.4

**TABLE 2:** Mortality rate for hepatitis C virus, hepatitis B virus and viral hepatitis – related hepatocellular carcinoma in England 2018 to 2024 applying the attributable fraction.

Year	2018	2019	2020	2021	2022	2023	2024
Population estimate (England)	55,924,528	56,230,056	56,325,961	56,554,891	57,144,395	57,932,470	58,620,101
HCC deaths	2507	2607	2661	2610	2628	2733	2770
**HCV as the likely cause of HCC**							
Attributable fraction	0.18065	0.18065	0.18065	0.18065	0.18065	0.18065	0.18065
Estimated number of death attributable to HCV	453	471	481	471	475	494	500
Estimated mortality rate per 100,000 population	0.81	0.84	0.85	0.83	0.83	0.85	0.85
**HBV as the likely cause of HCC**							
Attributable fraction	0.06452	0.06452	0.06452	0.06452	0.06452	0.06452	0.06452
Estimated number of death attributable to HBV	162	168	172	168	170	176	179
Estimated mortality rate per 100,000 population	0.29	0.30	0.30	0.30	0.30	0.30	0.30
**Viral hepatitis as the likely cause of HCC**							
Estimated number of death attributable to viral hepatitis	615	639	652	640	644	670	679
Estimated mortality rate per 100,000 population	1.10	1.14	1.16	1.13	1.13	1.16	1.16

Among the 28 individuals for whom HCV was the likely cause of their HCC, 15 also had alcohol reported as a possible cause and five MASLD. Whilst all individuals for whom their HCC was attributable to HCV were HCV antibody positive, only four were positive for HCV RNA or antigen indicative of an active infection. Thirty‐three per cent of the HCC cases in London were attributable to HCV, 15% for Oxford and Nottingham, 8% for Sheffield and 4% for Birmingham.

For individuals where HBV was the likely cause, one was coinfected with HCV, one with HDV and one also had MASLD reported; all were hepatitis B surface antigen positive. 12% of the HCC cases in Birmingham were attributable to HBV, 8% in London and 4% in Nottingham, Sheffield and Oxford.

Applying this estimate to the mortality envelope of individuals dying of HCC in 2024 (2770), 500 were estimated to be attributable to HCV and 179 were estimated to be attributable to HBV, equivalent to a crude mortality rate of 0.85 per 100,000 population and 0.30 per 100,000 population, respectively. Overall, 679 HCC deaths were estimated to be attributable to viral hepatitis equating to a crude mortality rate of 1.16 per 100,000 population (Table 2).

**FIGURE 1:**
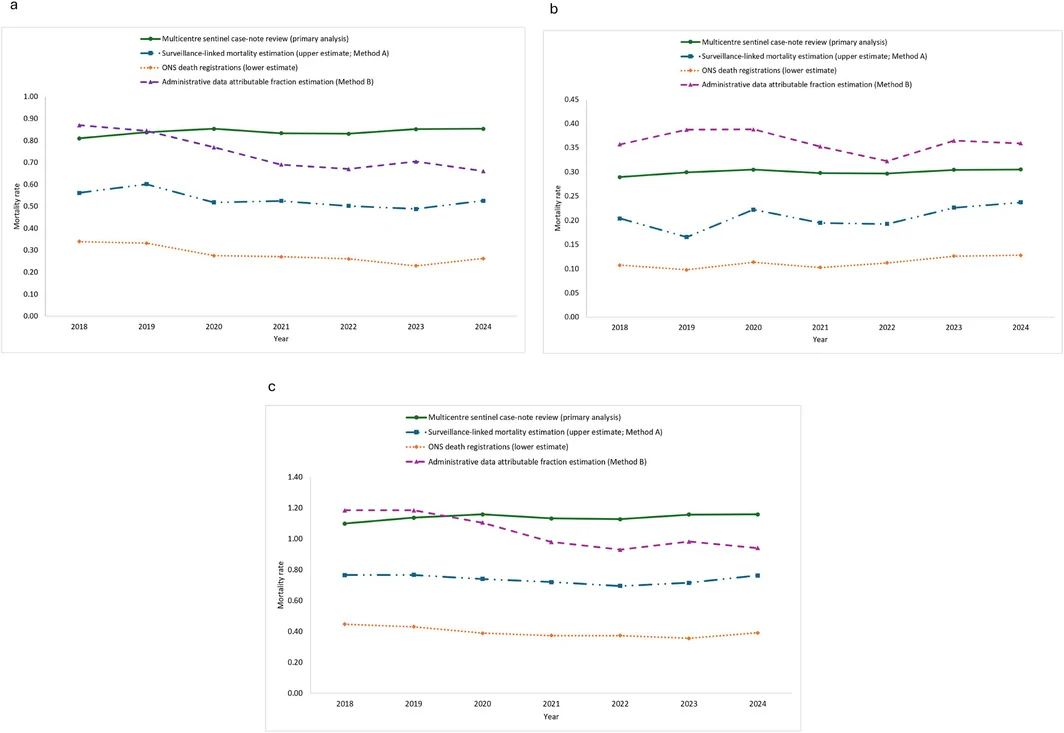
Mortality rate for (a) hepatitis C virus, (b) hepatitis B virus and (c) viral hepatitis–related hepatocellular carcinoma in England 2018 to 2024 by method of estimation.

Figure 1a–c shows mortality rates for HCC over time using the attributable fraction compared with mortality rates calculated by linking HCC death registrations with routine laboratory reports of hepatitis diagnoses and hospital episode statistics to flag an HCV and/or HBV diagnosis. Whilst the mortality rate for HBV estimated using the attributable fraction fell between the upper and lower estimate using surveillance methodology, for HCV, the attributable‐fraction‐generated mortality rate was higher than the upper estimate using surveillance methodology.

### 3.2 Attributable Fraction Estimates of Cirrhosis Decompensation due to Viral Hepatitis

The distribution of individuals with confirmed decompensated cirrhosis (DC) (127) can be seen in Table 3. Most had a likely cause of DC reported (125; 98.4%) and 109 (87.2%) had been screened for viral hepatitis. The median age was 63 years (interquartile range (IQR): 53–75), 56.2% (68/125) were male, and 77.7% (94/121) were of White ethnic origin. HCV was reported as the likely cause of DC for 1.6% (2/125) of individuals and HBV for 1.6% (2/125) of individuals. One of the individuals with DC attributable to HCV was also reported to have HCC and was therefore excluded as they were included in the HCC group. These data resulted in an estimated attributable fraction of 0.01 for HCV and 0.02 for HBV (Table 4).

**TABLE 3:** Demographics of individuals with decompensated cirrhosis (DC) attending six acute NHS trusts in England in 2024.

	*N*	%
DC shared	390	100
DC confirmed	127	32.6
DC with likely cause	125	98.4
**Sex**		
Male	68	56.2
Female	57	47.1
Unknown		
**Median age**	63 years (IQR: 53–75)	
**Ethnic group**		
Asian	14	11.6
Black	1	0.8
White	94	77.7
Mixed	1	0.8
Other	11	9.1
Unknown	4	
**Clinical main symptoms**		
Ascites	85	68.0
Jaundice	15	12.0
Encephalopathy	20	16.0
Bleed	18	9.5
Pleural effusion	2	1.1
**Site**		
Sheffield	16	12.8
Birmingham	25	20
Oxford	26	20.8
Nottingham	19	15.2
London	39	31.2
**HBV tests**		
HBV positive	1	0.9
HBV negative	108	99.1
**HCV tests**		
HCV Ab negative	104	95.4
HCV Ab positive	5	4.6
HCV RNA positive	2	1.8
**Likely cause of DC**		
HBV	2	1.6
*HBV only*	1	0.8
*With HDV*	1	0.8
HCV	2	1.6
*HCV only*	1	0.8
*With alcohol*	1	0.8
MASLD	27	21.6
Alcohol related	71	56.8
Other	24	19.2

**TABLE 4:** Mortality rate for hepatitis C virus, hepatitis B virus and viral hepatitis—related decompensated cirrhosis in England 2018 to 2024 applying the attributable fraction.

Year	2018	2019	2020	2021	2022	2023	2024
Population estimate (England)	55,924,528	56,230,056	56,325,961	56,554,891	57,144,395	57,932,470	58,620,101
DC deaths	4797	4978	5404	5384	5454	5479	6291
**HCV as the likely cause of DC**							
Attributable fraction	0.008	0.008	0.008	0.008	0.008	0.008	0.008
Estimated number of death attributable to HCV	38	40	43	43	44	44	50
Estimated mortality rate per 100,000 population	0.069	0.071	0.077	0.076	0.076	0.076	0.086
**HBV as the likely cause of DC**							
Attributable fraction	0.016	0.016	0.016	0.016	0.016	0.016	0.016
Estimated number of death attributable to HBV	77	80	86	86	87	88	101
Estimated mortality rate per 100,000 population	0.137	0.142	0.154	0.152	0.153	0.151	0.172
**Viral hepatitis as the likely cause of DC**							
Estimated number of death attributable to viral hepatitis	115	119	130	129	131	131	151
Estimated mortality rate per 100,000 population	0.206	0.212	0.230	0.228	0.229	0.227	0.258

Among the three individuals for whom viral hepatitis was the likely cause of their DC, one due to HCV and two due to HBV, the individual with HCV died during DAA treatment. Of the two cases with HBV, one was co‐infected with HDV and both were taking nucleot(s)ide treatment for HBV.

When this estimate was applied to the mortality envelope of individuals dying of DC in 2024 (6291), 50 of the deaths were estimated to be attributable to HCV and 101 were estimated to be attributable to HBV, equivalent to a crude mortality rate of 0.09 per 100,000 population and 0.17 per 100,000 population, respectively. Overall, 151 deaths due to DC were attributable to viral hepatitis, equating to a crude mortality rate of 0.26 per 100,000 population (Table 4).

**FIGURE 2:**
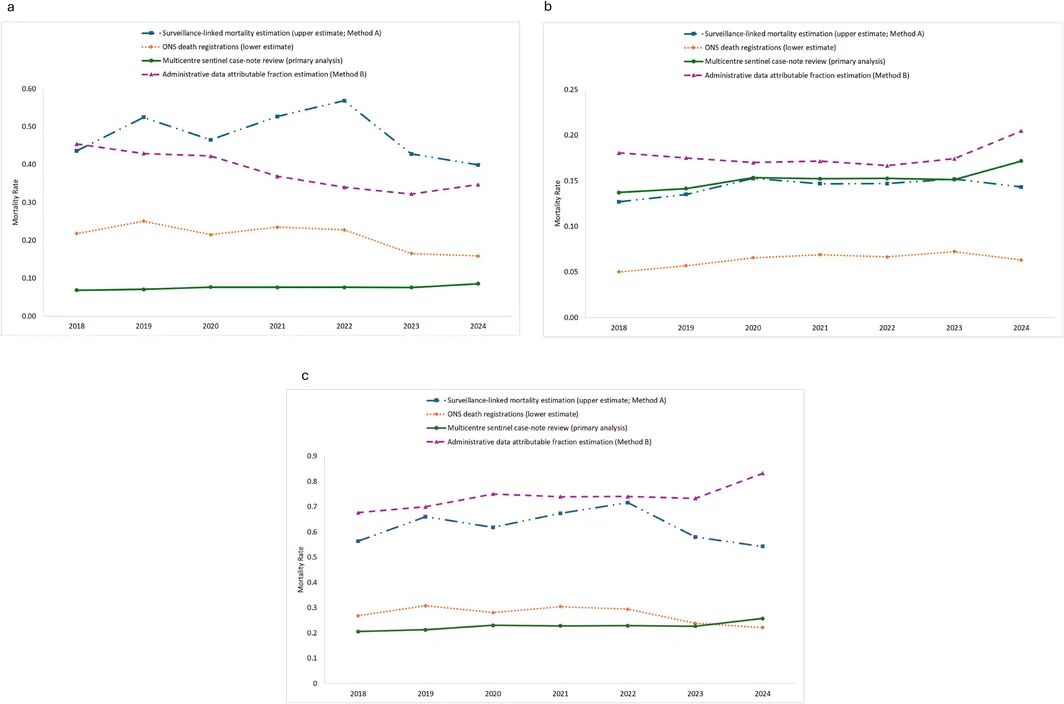
Mortality rate for (a) hepatitis C virus, (b) hepatitis B virus and (c) viral hepatitis–related decompensated cirrhosis in England 2018 to 2024 by method of estimation.

Figure 2a–c shows mortality rates for DC over time using the attributable fraction compared with mortality rates calculated by linking death registrations with routine laboratory reports of hepatitis diagnoses and hospital episode statistics to flag an HCV and/or HBV diagnosis. Whilst mortality rates for HBV estimated using the attributable fraction mirrored that of the upper estimate using surveillance methodology, for HCV the rate was below the lower estimate using surveillance methodology.

When all individuals ever testing positive for HCV (anti‐HCV positive) were included in the attributable fraction, the resulting mortality rates were similar to the upper estimates derived from surveillance‐based methods. However, when the definition was refined to include only individuals ever HCV positive with evidence of cirrhosis at the time of treatment, the estimated mortality rate was closer to the lower estimates obtained using surveillance methodology.

### 3.3 Viral Hepatitis Mortality

Using the attributable fraction estimates of HCC and DC the overall estimated viral hepatitis mortality for 2024 was 1.42 per 100,000 population, with an average of 1.37 per 100,000 between 2018 and 2024 (HCV‐related mortality: 0.94 per 100,000 in 2024; HBV‐related mortality: 0.48 per 100,000).

### 3.4 ICD‐10 Codes

Assessment of ICD‐10–based case identification against diagnoses confirmed through detailed case‐note review demonstrated variability in the positive predictive value of individual ICD‐10 codes for identifying decompensated cirrhosis. K72.0 (0%), R18.X (30.2%) and K72.9 (45.2%) had the lowest positive predictive values for identifying confirmed decompensated cirrhosis (Table 5).

**TABLE 5:** Positive predictive value of ICD‐10 codes used for identifying individuals with decompensated cirrhosis (only one ICD‐10 flag).

ICD‐10 code	Decompensated cirrhosis (case notes)				Total (*N*)
	No		Yes		
	*n*	%	*n*	%	
I85.0	5	50.0	5	50.0	10
I98.3	1	33.3	2	66.7	3
K70.4	6	42.9	8	57.1	14
K72.0	21	100.0	0	0.0	21
K72.1	0	0.0	1	100.0	1
K72.9	17	54.8	14	45.2	31
K76.7	0	0.0	2	100.0	2
R18.X	125	69.8	54	30.2	179
Total	175	67.0	86	33.0	261

### 3.5 Calculating the Attributable Fraction Using Established Healthcare Data

Between April 2023 and March 2024, 6715 individuals had an inpatient record in HES with an ICD‐10 code indicating HCC and 60,404 had codes consistent with evidence of DC.

Of the 6715 individuals with an inpatient HES record indicating HCC, 474 (7.1%) had an HBV diagnosis recorded through HES and 924 (13.8%) had an HCV diagnosis recorded in HES. An additional 45 individuals with HBV and 78 with HCV were identified through linking laboratory surveillance data on viral hepatitis diagnoses. The attributable fraction of HCC was estimated as 0.08 (519/6715) for HBV and 0.15 (1002/6715) for HCV. When applied to the mortality envelope, this corresponded to a crude mortality rate of 0.36 per 100,000 population for HBV‐attributable HCC and 0.70 per 100,000 population for HCV‐attributable HCC (Figure 1a–c).

For individuals with an inpatient HES record indicating evidence of DC (60,404), 932 (1.5%) had a HBV diagnosis recorded through HES and 1794 (3.0%) had a HCV diagnosis. An additional 181 individuals with HBV and 266 with HCV were identified through linking laboratory surveillance data on viral hepatitis diagnoses. The attributable fraction of DC was estimated as 0.02 (101/60,404) for HBV and 0.03 (187/60,404) for HCV. When applied to the mortality envelope, this corresponded to a crude mortality rate of 0.17 per 100,000 population for HBV attributable DC and 0.32 per 100,000 population for HCV attributable DC (Figure 2a–c).

Crude mortality rates over time calculated using established healthcare data to estimate the attributable fraction can be seen in Figures 1 and 2, alongside crude mortality rates from the case‐note review, and previously published estimates derived from death registrations linked to surveillance data.

## 4 Discussion

In collaboration with six hepatology centres in England, we undertook a case‐note review to estimate the attributable fraction for individuals with HCC and/or DC. Although the WHO generally recommends this approach where national surveillance data may not be reliable, our primary objective was to estimate HBV‐ and HCV‐attributable mortality using the WHO methodology. Secondary objectives were to assess the feasibility of implementing the WHO protocol in England and to compare the resulting estimates with those generated through England's existing surveillance systems, based on linked laboratory surveillance, hospital admissions and death registration data, to accurately monitor viral hepatitis‐related mortality on an ongoing basis. Using the WHO approach, the combined hepatitis B and C attributable mortality was estimated as 1.42 per 100,000 population, well below the WHO impact target of ≤ 6 per 100,000 for elimination. This corresponds to an estimated 830 deaths from HCC and DC attributable to hepatitis B and C in 2024, including 679 HCC deaths and 151 DC deaths. This is higher than estimates derived using England's established linkage‐based surveillance approach, highlighting the influence of case ascertainment and attribution methods on mortality estimates [3]. For hepatitis C the estimated mortality rate was 0.94 per 100,000 population (550 deaths combined, 500 HCC deaths and 50 DC deaths) and for hepatitis B, 0.48 per 100,000 population (280 deaths combined, 179 HCC deaths and 101 DC deaths) both below the WHO targets of ≤ 2 per 100,000 and ≤ 4 per 100,000 respectively. Among individuals with HCC, the likely cause was reported as HCV for 18.1% and HBV for 6.5%. Among individuals with DC the corresponding proportions were 0.8% and 1.6%, respectively.

While England has achieved the WHO mortality target, these figures demonstrate that viral hepatitis continues to contribute to severe disease outcomes. England's attainment of the elimination mortality threshold likely reflects a combination of public health interventions, including expanded HBV vaccination and antenatal screening programmes, increased testing and diagnosis, and broad access to effective antiviral treatment, particularly direct‐acting antivirals for HCV.

This multicentre sentinel case‐note review demonstrates that standardised review of clinical records across multiple regional centres is a feasible one‐off approach in a high income setting for estimating liver disease attributable to viral hepatitis. The feasibility is likely aided by the widespread use of electronic health records, well‐established specialist hepatology referral networks, routine clinical coding and access to linked national datasets. It can provide valuable clinical insight beyond routine surveillance data particularly where aetiology may be multi‐factorial and could be used to refine surveillance‐based estimates in the future though it was reassuring that the case‐notes‐based estimate was similar to the estimate generated through applying the same methodology to routine healthcare data. However, a case‐notes review is resource‐intensive which may be a challenge to replicate particularly in resource‐limited settings and is unlikely to deliver added value as a routine method for estimating viral hepatitis‐related mortality where robust surveillance systems are in place. It relies on strong clinical networks and is constrained by several limitations in addition to the significant resource requirement. Data completeness is variably affected by the hub‐and‐spoke service model, in which patients receive care across both local referring hospitals and specialist tertiary centres. Consequently, key clinical information is often fragmented across referring and treating institutions, leading to missing demographic and clinical variables, particularly among referred HCC patients. Additionally, while a standardised protocol was used, the attribution of aetiology relies on clinical judgement and the interpretation of often incomplete records, introducing inter‐observer variability (“human factor”), especially in patients with multiple risk factors. Consequently, although a case‐note review supports robust population‐level estimates, it is less reliable for detailed comparisons across sites or subgroups, where findings may be influenced by differences in data availability and subjective interpretation.

HCV remains a major driver of both HCC and decompensated cirrhosis cases in England. This is despite the successful roll‐out of DAAs which led to a reduction in the prevalence of HCV and a decrease in both ESLD and HCC mortality associated with HCV [14]. This might represent cases where treatment came too late to prevent disease progression. This is consistent with global evidence from a recent systematic review and meta‐analysis, which demonstrated that a substantial proportion of hepatocellular carcinoma cases are attributable to hepatitis B and C infections, alongside other metabolic and alcohol‐related risk factors [2]. Previous research showed that the mortality decreases for ESLD came much earlier than those for HCC, likely due to the early prioritisation of individuals with advanced liver disease, which had an impact on their mortality risk but did not remove the risk of progression to liver cancer [4]. This emphasises the importance of early diagnosis and treatment which are critical to ensuring continued progress towards HCV elimination.

Nearly one in five individuals with HCC had a disease attributable to HCV, including both current and past infections. In contrast, for DC, HCV was identified as the likely cause through the case‐note review only among individuals with active HCV infection. This highlights that whilst DAAs will result in a cure, individuals with advanced liver disease are still at risk of developing HCC. A meta‐analysis reported a pooled HCC incidence of 0.5 per 100 person‐years among individuals with F3 fibrosis [15]. For individuals with cirrhosis, the pooled incidence was 2.1 per 100 person‐years, with the HCC risk decreasing with each additional year of follow‐up after HCV cure. The authors concluded that universal screening of patients with F3 fibrosis was not cost effective, with EASL clinical practice guidelines suggesting surveillance be considered based on individual risk assessment [16]. All individuals with HCC attributable to HCV had evidence of cirrhosis at the time of treatment.

Where viral hepatitis was identified as the likely cause of HCC, individuals with HCV were more likely to have additional risk factors, such as alcohol and MASLD, compared with those HBV. A study in China [17] reported that the risk of HCC was 2.61‐fold higher in individuals with HCV and concomitant alcohol‐related liver disease compared with individuals with HCV alone and 2.01‐fold higher when comparing for HBV. Similarly, Gonzalez‐Aldaco et al. [18], highlighted that although MASLD and HCV are independently associated with liver damage, their coexistence accelerates liver disease progression, and that clinical follow‐up should be implemented for all HCV patients after viral eradication.

Attributable fractions have been estimated in Bulgaria, Portugal and Norway [10]. In all three countries, cases were identified from a single health facility, and the estimated attributable fractions were higher than those we report for England. In Norway and Portugal, HCV accounted for approximately one‐quarter of cirrhosis cases, whereas in Bulgaria HBV was predominated. A similar pattern was observed for HCC, with HCV associated with more than one‐third of cases in Norway and Portugal, while HBV was the more common underlying cause in Bulgaria. This work was undertaken to assess the feasibility of implementing the protocol developed by the WHO to support estimating attributable fractions, which raised several challenges, specifically the time and resources needed to review hospital records. To mitigate the impact on services, hospital inpatient records using ICD‐10 codes were used to identify patients requiring note reviews; however, this resulted in challenges related to the sensitivity of ICD‐10 codes for predicting cirrhosis. ICD‐10 codes R18 (*ascites*) and K72.0 (*acute and subacute hepatic failure*) demonstrated the lowest sensitivity for identifying cirrhosis, likely because these codes represent clinical manifestations or acute syndromes that occur across a broad range of conditions, not exclusively cirrhosis. Nevertheless, R18 could not be excluded from the algorithm, as it correctly identified cirrhosis in approximately one‐third of patients for whom only a single ICD‐10 code was recorded. Similar findings are reported by Burkholder et al. [19] with a 51% positive predictive value among those with confirmed cirrhosis, and Khalifa et al. [20] reporting a sensitivity of 42.9% for R18 identifying cirrhosis. In the absence of a cirrhosis register, further work is needed to refine how to best identify those with cirrhosis and differentiate those with decompensated cirrhosis through surveillance.

Through this work, we can be reasonably confident that our surveillance methods produce estimates that broadly reflect viral‐hepatitis‐related mortality. However, there are important considerations and areas for further refinement. The mortality rates calculated using the attributable fraction through the case‐note review for hepatitis B were similar and/or fell within the upper and lower mortality rates calculated using surveillance data [4]. In contrast, for hepatitis C, the mortality rates estimated through the case‐note review for individuals with HCC were higher than our highest estimate using surveillance data, and for individuals with DC, the HCV mortality rate was lower than our lowest estimate. The difference for HCV‐related DC mortality rates is likely due to the surveillance method using any evidence of HCV, whereas, via the case‐note review, HCV was only identified as the likely cause of DC for individuals with an active infection. When including all individuals with any evidence of HCV, the mortality rate was similar to our upper estimate, and including those with evidence of cirrhosis at the time of HCV treatment, the mortality rate was closer to our lower estimate. For viral‐hepatitis‐related HCC, mortality estimates using the attributable fraction were higher than those obtained through the linkage of surveillance data to ONS death registrations. While it is difficult to fully disentangle the reasons for this discrepancy, several factors are likely to contribute, including the underreporting of cases within surveillance systems used for linkage and the lower but continued risk of HCC following treatment [21]. Through this work, we can be confident that adapting our methods that the estimates produced through surveillance data will reflect viral hepatitis mortality rates.

The study has several limitations. Firstly, the study population was recruited based on the capacity of centres to participate, which may have introduced selection bias, and it is difficult to determine how representative the six included sites are of England as a whole. Secondly, the study relied on administrative data to identify individuals with the sequelae of interest. As these data are not collected for research purposes, they have some well‐recognised limitations, including missingness. This may have introduced selection bias, for example if an ICD‐10 code for HCC or decompensated cirrhosis was not recorded, and misclassification bias, for example if an ICD‐10 code for viral hepatitis was missing. Data linkage also depended on the completeness of key patient identifiers, meaning that linkage errors could have biased the results. Additionally, the analysis applied a single attributable fraction estimate across all years presented, which may not reflect true temporal trends, as it assumes no change over time; however, published mortality data suggest that HBV‐ and HCV‐related mortality rates have remained relatively stable over this period. Finally, a high proportion of study participants had an unknown hepatitis status, particularly among those included due to decompensated cirrhosis. This likely biased the findings and highlights potential gaps in routine testing.

While England reports a lower fraction of viral‐hepatitis‐attributable liver disease than other European countries where studies have been conducted, viral hepatitis continues to contribute significantly to the national burden of HCC and cirrhosis. The successful impact of DAAs is evident, but the continued prevalence of these sequelae emphasises that treatment must be paired with earlier detection to prevent irreversible liver damage. Furthermore, our results validate the use of linked administrative data as a viable, scalable method for future estimation exercises.

## Author Contributions

D.E. developed the protocol and received REGG approval. A.P. and D.E. managed the data and liaised with the sites. P.T., J.P., A.B., M.L., S.R., S.M., E.P., A.B., L.H., M.P., A.E. all submitted data from their sites and provided insights to the results and discussion. D.E., A.P., R.S. undertook the analysis and had access to the complete dataset. D.E., A.P., R.S. drafted the manuscript. M.D., R.S. had senior oversight of the work. M.D., R.S., C.S. reviewed and provided input into the manuscript. All authors reviewed and approved the manuscript submission.

## Funding

This work was supported by the National Institute for Health and Care Research.

## Conflicts of Interest

Paul M Trembling has received honoraria from Gilead Sciences.

## Supporting information


**Data S1:** jvh70228‐sup‐0001‐Supplementary Materials.docx. **Section S1:** Data extraction report form.
**Section S2:** Sensitivity analysis.
**Table S1:** Characteristics of the Methods Used to Estimate HBV‐ and HCV‐Attributable Mortality.

## Data Availability

The data that support the findings of this study are available from the corresponding author upon reasonable request.
