# Supplementary material for: Estimating the Fraction of Hepatocellular Carcinoma and Decompensated Cirrhosis Attributable to Viral Hepatitis in England Using Electronic Health Records and Case‐Notes Review
Source: J Viral Hepat. 2026 Sep 21;33(10):e70228. doi: 10.1111/jvh.70228 (PMC13594983; doi:10.1111/jvh.70228)
Supplement: Supplementary file 1 [file JVH-33-0-s001.docx]

**Supplementary materials**

**Section S1: Data extraction report form.**

**Section S2: Sensitivity analysis**

Table S1: Characteristics of the Methods Used to Estimate HBV- and HCV-Attributable Mortality.

| **Method** | **Purpose** | **Data source** | **HBV/HCV definition** | **Mortality estimate** |
| --- | --- | --- | --- | --- |
| WHO AF case-note review  (main analysis) | Primary analysis | Case-note review at 6 hepatology centres + ONS mortality envelope | Clinical review of HBV/HCV status and likely cause | AF × HCC/DC deaths |
| Surveillance-linked mortality  (Method A) | Comparison analysis | ONS deaths registrations + hepatitis laboratory surveillance + Hospital admission data (Inpatient, Outpatient). | Laboratory-confirmed HBV/HCV diagnosis and/or HBV/HCV recorded in linked datasets | Direct count of linked HBV/HCV-attributable deaths |
| Administrative AF (Method B) | Comparison analysis | Hospital admission (Inpatient, Outpatient and accident and emergency) + Hepatitis laboratory surveillance | HBV/HCV recorded alongside a hospital admission and/or linked to a laboratory-confirmed HBV/HCV diagnosis | AF derived from routine data × HCC/DC deaths |

ONS: Office for National Statistics
